# Right axillary lymph node metastasis of carcinoma of the cecum with histologically proven cutaneous lymphatic invasion by carcinoma cells: a case report

**DOI:** 10.1186/s40792-015-0054-0

**Published:** 2015-06-17

**Authors:** Yutaka J Kawamura, Michitaka Kohno, Junji Shiga, Naoki Asakage, Minoru Hatano, Hirohisa Okame, Junichi Sasaki, Shoichi Tobari, Katsunori Nishida

**Affiliations:** Department of Surgery, Tsudanuma Central General Hospital, 1-9-17 Yatsu, Narashino-shi, Chiba 275-0026 Japan; Department of Pathology, Tsudanuma Central General Hospital, 1-9-17 Yatsu, Narashino-shi, Chiba 275-0026 Japan

**Keywords:** Colorectal carcinoma, Lymphatic invasion, Axillary lymph node metastasis

## Abstract

Axillary lymph node metastasis from colorectal carcinoma is extremely rare, and this scarcity hinders understanding of its pathogenesis and, thus, the application of appropriate management. Here, we present a case with axillary lymph node metastasis of cecal carcinoma associated with macroscopic invasion of the skin of the abdominal wall with histological evidence of such invasion, findings which support our hypothesis that the axillary lymph node metastasis developed via the lymph channels in the skin of the abdominal wall. A 76-year-old woman with cecal carcinoma (T4N1M0), complicated with an abdominal wall abscess, underwent right hemicolectomy with partial resection of the abdominal wall. Histology demonstrated multiple sites of lymphatic invasion in the skin. Two months later, an enlarged right axillary lymph node was noticed on CT, and an excisional biopsy was obtained, which later confirmed metastatic adenocarcinoma. This is the first case report of axillary lymph node metastasis of carcinoma of the cecum with histologically proven invasion via the lymphatic system in the skin. If axillary lymph node metastasis results from aberrant lymphatics due to invasion from an adjacent organ, and not the result of systemic malignant disease, it may be considered as a surgically curable pathology. Therefore, the authors advocate that patients with axillary lymph node metastasis should be evaluated with regard to the possibility of surgical curability.

## Background

Axillary lymph node metastasis of colorectal carcinoma is extremely rare. There are only a few reports in the English literature regarding this phenomenon, and all of them are single case reports [1–4]. Adequate management of such metastasis has not been established, because of the scarcity of the condition and our lack of knowledge as to why such distant nodes are involved. Here we report the first case of axillary lymph node metastasis of a cecal carcinoma with histologically proven cutaneous lymphatic invasion, in which case, we believe may give us insight into this uncommon pathogenesis.

## Case presentation

A 76-year-old woman presented with abdominal pain, fever, and a color change in the skin of the abdominal wall. Two weeks before presentation, the patient noticed abdominal pain and fever accompanied with a loss of appetite. Then, gradually, the skin color of the right lower quadrant of the abdomen darkened with deterioration of her general condition. Her medical history included surgeries for dislocations of the hip joint.

**Fig. 1:**
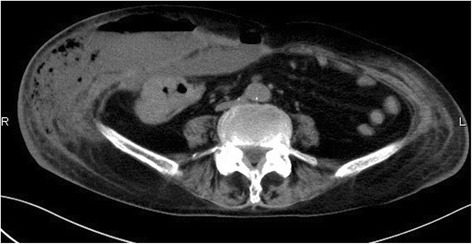
CT demonstrating a large abdominal wall abscess containing air. There was no apparent connection between the abdominal wall abscess and the abdominal cavity, although the wall of the ascending colon adjacent to the abscess was thickened

Physical examination revealed a body temperature of 37.8 °C, blood pressure of 106/70 mmHg, and heart rate of 129 beats/min. The skin of the right lower abdominal wall, 8 × 3 cm in size, was black and associated with fluctuation and tenderness, indicating an abdominal wall abscess complicated by skin necrosis. The white blood cell count was 19,000/m^3^ and CRP, 46.04 mg/dl. Laboratory data were otherwise nonspecific except for mild dehydration. Serum CEA (normal range; 0–5 ng/ml) and CA19-9 (normal range; 0–37 U/ml) were 1.2 ng/ml and less than 2 U/ml, respectively. Emergent CT was performed and demonstrated a large abdominal wall abscess of 15 × 12 cm in size, which contained a moderate amount of air (Fig. 1). There was no apparent connection between the abscess and the abdominal cavity, although the ascending colon and the cecum were adjacent to the abscess; though inconclusive at that time, moderate thickening of the colonic wall was noticed.

Emergent surgery was carried out with the preoperative diagnosis of abdominal wall abscess which was derived from the unconfirmed pathology in the adjacent large intestine. The necrotic skin was extirpated, and a huge amount of pus was drained via three additional skin incisions. The abscess cavity was then intensively irrigated with normal saline, and three drains were inserted subcutaneously. There was no connection seen between the abscess and the abdominal cavity. The results of a bacteriological study demonstrated the presence of Escherichia coli and Streptococcus pyogenes.

Postoperatively, the patient was transferred to the intensive care unit where she was treated for sepsis and respiratory failure. During the postoperative period, which was also complicated by a brain infarction, no fecal material was drained from any of the drains.

**Fig. 2:**
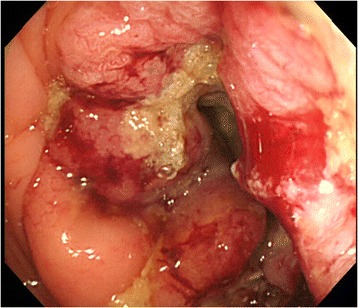
Colonoscopy revealing an ulcerated irregular tumor in the cecum

**Fig. 3:**
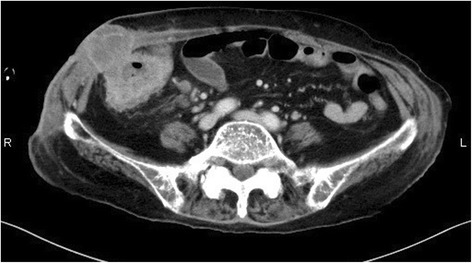
CT performed 39 days after the initial surgery demonstrating a mass that had originated from the ascending colon and had invaded the abdominal wall

Thirty-nine days after the initial operation, when the patient was considered to tolerate both mechanical bowel preparation and the examination itself, a colonoscopy was performed. An ulcerated irregular tumor was found in the cecum (Fig. 2). Biopsy revealed a well-differentiated adenocarcinoma. At this time, a CT scan showed a mass that originated from the cecum that invaded into the abdominal wall at the exact site where the wall thickening had been pointed out before the first operation (Fig. 3). There was no evidence of distant metastasis. MRI revealed no brain metastasis. Serum CEA and CA19-9 were 5.7 ng/ml and less than 2 U/ml, respectively. Although several lymph nodes were detected in the bilateral axillary area, because of their size being 1 cm or less, we considered them as nonspecific.

**Fig. 4:**
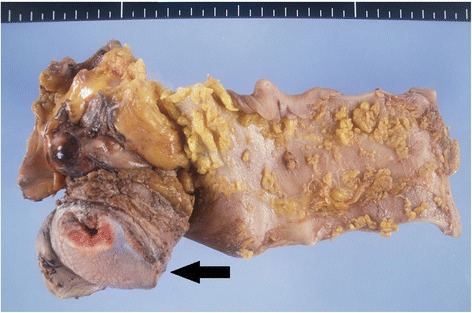
Resected specimen. En bloc resection was carried out for carcinoma of the cecum with abdominal wall invasion (*black arrow*)

**Fig. 5:**
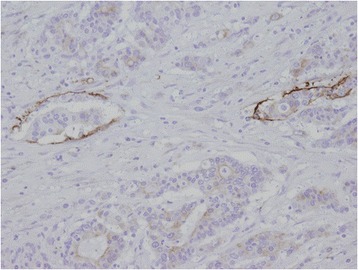
Immunohistochemical study using D2-40, revealing invasion of carcinoma cells into the lymphatic channels of the skin

Oncological right hemicolectomy with partial abdominal wall resection was performed with macroscopically curative intent (Fig. 4). Histological examination revealed a well-differentiated adenocarcinoma (T4N1M0) with skin invasion. Proximal and distal margins of the resected specimen were negative; nevertheless, the surgical margin around the site of skin invasion was considered to be pathologically positive for carcinoma. Detailed examination of the skin proved the presence of multiple sites of lymphatic invasion (Fig. 5). The postoperative course was uneventful, and adjuvant chemotherapy was declined by the patient.

**Fig. 6:**
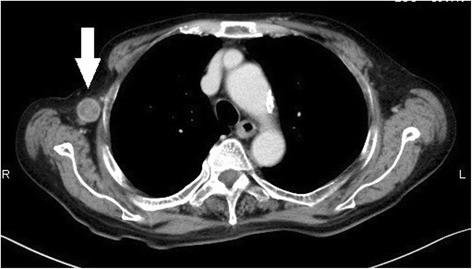
CT demonstrating an enlarged lymph node of 3 cm in diameter in the right axillary region (*white arrow*)

**Fig. 7:**
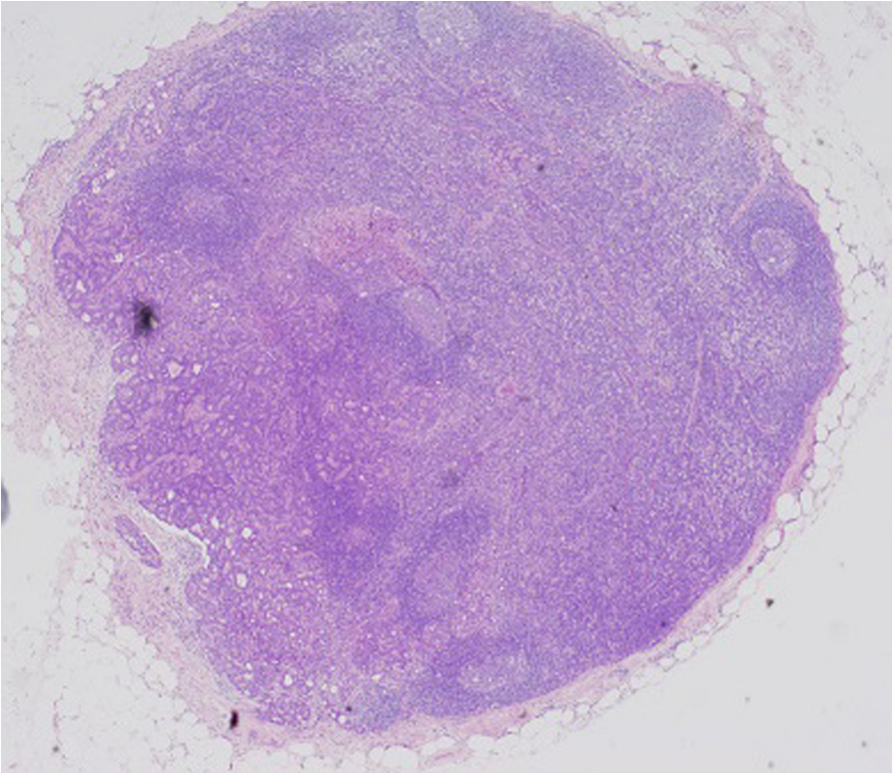
Histological examination of the enlarged right axillary lymph node, proving the presence of metastatic adenocarcinoma

Two months after the hemicolectomy, a CT was done for the sake of surveillance, and an enlarged lymph node of 3 cm in diameter was detected in the right axillary region (Fig. 6). There was no distant metastasis. Physical examination and ultrasonography of the breast revealed no mass in bilateral breasts. Serum CEA and CA19-9 were 1.9 ng/ml and less than 2 U/ml, respectively. Excisional biopsy of this axillary lymph node was done, and histological examination revealed metastatic adenocarcinoma (Fig. 7).

Twenty-one days later, a local recurrence in the abdominal wall, which had also been detected on CT, performed 2 months after the initial operation, was resected with curative intent. At the same time, systematic axillary lymph node dissection was performed, because CT had showed several lymph nodes in the right axillary region. Twenty nodes were dissected, and histological examination revealed no cancer involvement. Adjuvant chemotherapy was again proposed and declined. The patient was then put on surveillance.

## Discussion

Management of axillary lymph node metastasis of colorectal carcinoma is a clinical challenge. To the best of our knowledge, there are only four reports on this phenomenon in the English literature [1–4]. Of these four cases, two of them were those of solitary axillary lymph node metastasis [1, 2], and one was associated with metastasis to both the cervical and axillary lymph node [3]. In the remaining case, breast and ipsilateral axillary lymph node metastasis had developed [4].

With regard to the etiology of axillary lymph node metastasis of colorectal carcinoma, the last case cited suggests that the axillary lymph node metastasis may have developed from the site of the breast metastasis, which is considered as hematogenous metastasis, via a mechanism similar to that operating in primary breast carcinoma. In cases without breast metastasis, the hypothesis is that the axillary lymph node metastasis occurs via cutaneous lymphatic channels in the abdominal wall, whose channels eventually proceed in the axillary lymph nodes [5].

This is the first report of a case with axillary lymph node metastasis of colorectal carcinoma accompanied by histologically proven cutaneous lymphatic invasion. Initially, the patient presented with an abdominal wall abscess. Although emergent surgery did not reveal any connection between the abscess and peritoneal cavity, we consider that the primary pathology was a cecal carcinoma associated with perforation, which spontaneously sealed and developed into an abdominal wall abscess. We propose that at that time, carcinoma cells invaded into the abdominal wall, whose invasion enabled them to make contact with and enter the uncommon lymphatic channels, which, in this case, would have been the channels in the skin of the abdominal wall. We believe that this report provides histological evidence supporting the hypothesis that the axillary lymph node metastasis seen in the present case spread via these cutaneous lymphatic channels.

This case also emphasizes the importance of the systematic surveillance of patients who have undergone potentially curative resection of colorectal carcinoma, especially those with adjacent organ invasion, which may associate with lymphatic channels different from anatomical and physiological lymphatic channels of the primary site.

Given that axillary lymph node metastasis develops via the cutaneous lymphatic channels, what would be adequate treatment? If axillary lymph node metastasis is a pathology that should be considered as systemic, the treatment of choice would be systemic chemotherapy. However, metastasis along the uncommon lymphatic channels could be handled as a local disease, like usual lymph node metastasis seen along the arteries supplying the corresponding site of the colon. With regard to the long-term outcome, the observation period of our case was too short to permit adequate discussion. Three cases out of the above-cited four cases died within 2 years after the diagnosis of axillary lymph node metastasis. While the fourth case was alive without recurrence at the time of that report, the observation period was only 1 year. Therefore, according to the findings reported in the English literature, the long-term outcome of patients with axillary lymph node metastasis is considered as poor. However, in the Japanese literature, we found three cases with axillary lymph node metastasis of colorectal cancer who survived more than 3 years after the diagnosis or surgery without recurrence [6–8]. Therefore, if potentially curative resection is possible, surgery may be the treatment of choice in the hope that the axillary lymph node metastasis is a curable metastasis that occurred solely via different lymphatic channels. Recent advances in genetic/epigenetic research have revealed scores of prognostic factors in colorectal carcinoma. The authors hope that such analysis may contribute to the decision-making in patients with uncommon presentations like this case in near future.

## Conclusions

A case of axillary lymph node metastasis from a cecal carcinoma associated with abdominal wall invasion was reported. In this case, invasion of the carcinoma cells into the cutaneous lymphatic vessels was histologically documented, findings which the authors believe provide supportive evidence for the hypothesis that axillary lymph node metastasis can develop via cutaneous lymphatic channels and not necessarily be a sign of systemic disease. This hypothesis may underscore the possibility of long-term survival of selected patients after potentially curative resection of axillary lymph nodes.

## Consent

Written informed consent was obtained from the patient for publication of this case report and any accompanying images. A copy of the written consent is available for review by the Editor-in-Chief of *Surgical Case Reports*.

## Abbreviations

CT: computed tomography
